# Assessment of the Use of Telemedicine by Brazilian Otolaryngologists through an Online Questionnaire

**DOI:** 10.1055/s-0044-1786385

**Published:** 2024-05-25

**Authors:** Alexandre Wady Debes Felippu, Thiago Picolli Morsch, Deusdedit Brandão Neto, Eduarda Montenegro Moretti, André Wady Debes Felippu, Filippo Cascio, Richard Louis Voegels

**Affiliations:** 1 Department of Otorhinolaryngology, Hospital das Clínicas, Faculdade de Medicina, Universidade de São Paulo, São Paulo, SP, Brazil; 2 Department of Otorhinolaryngology, Instituto Felippu, São Paulo, SP, Brazil; 3 Department of Otorhinolaryngology, Azienda Ospedaliera Papardo, Messina, Italy

**Keywords:** telemedicine, COVID-19, otorhinolaryngology, technology

## Abstract

**Introduction** The coronavirus disease 2019 (COVID-19) pandemic has prompted a transformation in medical practice, including the adoption of telemedicine in Brazil and globally. Otorhinolaryngology, a field at high risk of viral transmission, has witnessed an increasing use of telemedicine tools. However, the extent and challenges of telemedicine in this field in Brazil are not well understood. In the present study, we applied a questionnaire to Brazilian otorhinolaryngologists during the pandemic to assess telemedicine's advantages and challenges, shedding light on its integration and persistent issues in the field.

**Objective** To assess the usage profile of telemedicine in the clinical practice of otolaryngology in Brazil.

**Methods** The present was a cross-sectional observational study with convenience sampling. It was conducted as a web-based questionnaire distributed and advertised to Brazilian otorhinolaryngologists through social media posts on WhatsApp (Meta Platforms, Inc., Menlo Park, CA, USA), Instagram (Meta Platforms, Inc.), Facebook (Meta Platforms, Inc.), as well as direct messaging and email.

**Results** The sample size was 186 participants. A total of 69% of them had already provided or were currently providing telemedicine services, and 34% considered it a frequent or very frequent form of work at the time of data collection. In total, 90% of the respondents considered the lack of physical examination a problem in otolaryngological teleconsultations, while 64% reported frequently or very frequently resolving patient problems through teleconsultations.

**Conclusion** Telemedicine emerged in the context of the COVID-19 pandemic as a promising tool for remote patient care. More studies are needed to elucidate its role in the context of limited physical examination.

## Introduction

The public health problem caused by the coronavirus disease 2019 (COVID-19) pandemic in Brazil and worldwide has forced traditional medical practice to reinvent itself. After the publication of ordinance no. 467 in March 2020 by the Brazilian Ministry of Health, electronic medical consultations were given legal support. With advances in communication technology, the use of telemedicine has become a reality in various fields. 1 With the emergence of the pandemic, telemedicine has become crucial in mitigating the transmission of the virus. This is particularly important in a pandemic scenario, as the safety of healthcare professionals is essential to ensure that attention to the population is not compromised by a lack of healthcare professionals in the field. 2 3

Otorhinolaryngology is one of the specialties with the highest susceptibility to transmission due to intimate contact with the patient's airway; therefore, tools that can prevent viral dissemination have been widely used. 4 5 6 In Brazil, little is known about the scale and ways in which telemedicine has been applied in this field. Many obstacles still hinder the use of technology for medical consultations. In this scenario, it is essential to conduct studies that aim to understand the relevance, applicability, and difficulties of using telemedicine in our country.

To this end, in the present study, we applied a questionnaire to otorhinolaryngologists working in Brazil to assess the use of telemedicine, particularly during the COVID-19 pandemic, with the aim of elucidating the primary advantages and challenges encountered when implementing this technology. The findings of the present investigation have the potential to delineate areas in which telemedicine has already been integrated into otorhinolaryngology, while also elucidating the persisting issues that our field continues to encounter when implementing this tool.

## Objective

To assess the usage profile of telemedicine in the clinical practice of otolaryngology in Brazil.

## Methods

### Design

Cross-sectional observational study with level IV of evidence.

### Sampling

Convenience sampling.

### Study Population and Data Collection Instruments and Techniques

The study population consisted of physicians who provide otorhinolaryngologic care in Brazil. Recruitment was exclusively electronic, through social media posts on WhatsApp (Meta Platforms, Inc., Menlo Park, CA, United States), Instagram (Meta Platforms, Inc.), Facebook (Meta Platforms, Inc.), as well as direct messaging and email. The questionnaire was applied between July 20th and September 23rd, 2021. A recruitment text was written and sent.

The questionnaire was developed by the researchers using dichotomous questions, Lickert scale, and open field. The survey took approximately five minutes to complete.

### Inclusion Criteria

Physicians who provide otorhinolaryngology care.

Participants who filled out the free and informed consent form.

### Exclusion Criteria

Researchers and collaborators of the study.

### Risks

Patients were not exposed to new biological risks since the study was based on data collection through a questionnaire, as aforementioned.

## Results

The present study included 186 (64% of male and 36% of female) participants. A total of 66% of the professionals reported working in capital cities, 26%, in rural areas, and 8%, in both, with São Paulo being the state with the largest number of participants, accounting for 52% of the study's sample. Most participants (58%) reported being in the age range between 30 and 45 years ( Table 1 ).

**Table 1:** Sex, age, and place of practice of the respondents

	Percentage
**Sex**	
Male	64%
Female	36%
**Age**	
< 30 years	7%
30–45 years	58%
46–60 years	21%
> 60 years	14%
**Place of practice**	
Capital city	66%
Countryside	26%
Both areas	8%

General otolaryngology was the most common field of work reported in the questionnaire (64%), followed by rhinology (18%), and otology (7%). A total of 70% of the participants cared for patients in private clinics, and 38%, in teaching or academic hospitals. As a source of income, 78% reported both health insurance plans and private patients. Only 35% reported being paid by the public health system.

A total of 69% of the participants reported that they had already provided or were currently providing telemedicine services, with 48% working in this sector for over a year. At the time of data collection, 34% of the participants considered telemedicine a frequent or very frequent form of work.

Private patients were the group that mostly sought care through teleconsultation, with 67% of physicians reporting the provision of care to this group by teleconsultation at least occasionally. A total of 84% of physicians who were telemedicine users reported never having provided care to patients from the public health system using this service model.

Overall, 42% of the otolaryngologists interviewed reported conducting their teleconsultations in private clinics, 19%, in clinics, and 38%, at home. The most used digital tools were WhatsApp (46%), electronic medical records (22%), Zoom (20%), and Google Meet (11%). Only 14% did not consider themselves proficient in handling software and technological resources for telemedicine.

Only 13% of the interviewed physicians reported frequently or very frequently attending telemedicine consultations with patients from other states.

**Graph 1:**
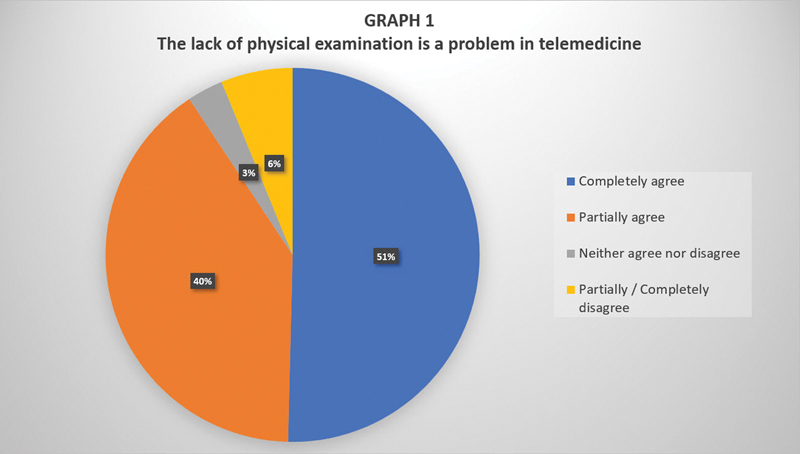
Rate of respondents who considered the lack of physical examination a problem in telemedicine.

The use of telemedicine for first-time consultations, follow-up, and check-ups was reported by 73%; 66% indicated that they did not use any screening methods during these consultations; and 90% of the respondents considered the lack of physical examination a problem in otolaryngological teleconsultations ( Graph 1 ), but only 68% reported the performance of another kind of examination.

Among the 31% of respondents who had never provided or did not provide telemedicine consultations, the most common reason (25%) was that physicians did not believe in consultations without a physical presence. On the other hand, lack of access to technology and difficulty in dealing with it were mentioned as reasons only by 4% and 1% of the sample respectively.

Regarding telemedicine payment, 60% of respondents reported charging the same amount for telemedicine and in-person consultations, with only 20% feeling completely satisfied with the remuneration for virtual appointments.

Regarding accessibility, only 33% considered the adaptation to the use of telemedicine extremely easy, and the main difficulties reported were the patient's poor internet connection (30%), the patient's difficulty in dealing with technology (24%), and the patient's lack of access to technology (11%). The lack of adequate institutional infrastructure was mentioned as a complaint only by 8% of telemedicine users.

A total of 75% of respondents partially or fully agreed that telemedicine is a useful tool, with only 28% citing insecurity in its use. One fifth (20%) of the study's subjects reported feeling uncomfortable or embarrassed to conduct consultations via telemedicine.

**Graph 2:**
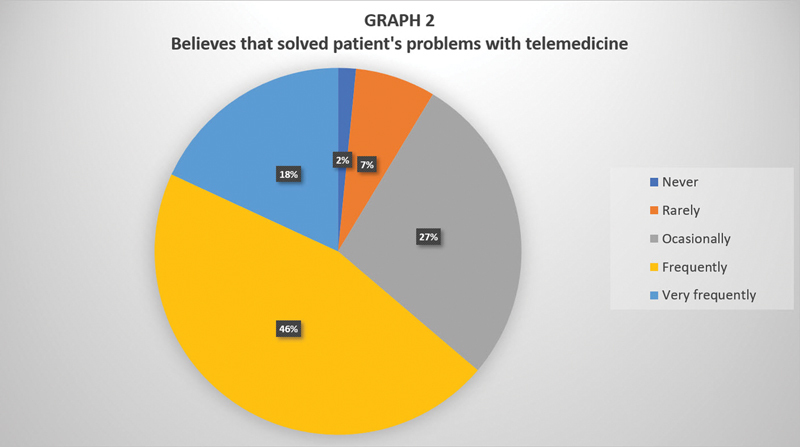
Rate of respondents who believe that they have solved their patients' problems through telemedicine.

Among the otolaryngologists who are proponents of telemedicine, 64% reported frequently or very frequently resolving patient's problems through teleconsultations ( Graph 2 ).

More than half of those surveyed (63%) believed that patients are supportive of telemedicine, even though no questionnaires have been specifically administered to this population.

Finally, 3/4 (75%)of the respondents indicated their intention to continue with teleconsultations after the pandemic, but only 27% planned to do so frequently or very frequently; the majority (41%) were interested in maintaining it only occasionally.

## Discussion

The present is one of the first studies to evaluate the use of telemedicine among otolaryngologists practicing in Brazil. The COVID-19 pandemic has caused significant changes in healthcare, accelerating processes that may have taken many years to be put into practice. 5 In light of the difficulties imposed on the contact between physician and patient, the use of telemedicine in Brazil was temporarily approved in March 2020 by the Brazilian Ministry of Health and a law on the subject was enacted by the Brazilian Federal Government in December 2022 (law no. 14.510). Technological advances have resulted in greater security regarding medical records. Video call tools have multiplied. The speed of the internet is becoming increasingly faster, greatly facilitating the use of such technology. 7

The protection of the health of the patient and the physician has indeed become very relevant in the face of a scenario of widespread viral transmission. Otorhinolaryngology, as well as other fields, has a higher level of exposure. 8 9

The results of the present study point to relevant information regarding the use of this type of tool. It is worth noting that the sample presents selection biases. The convenience sample collected through digital means may exclude a portion of otolaryngologists who are not yet adept at using digital tools.

According to the 2012 census of the Brazilian Association of Otolaryngology, Brazil had approximately 7 thousand registered professionals. Only 186 responded to the survey, which indicates a population that may not represent the entirety of physicians in this field.

Lack of access to technology and difficulty in dealing with it were mentioned as reasons only by 4% and 1% of the sample respectively. It is necessary to consider, however, that the recruitment for the present study might have given preference to physicians who already had a closer relationship with technology, as the sample was recruited through online platforms. This fact could justify such a low rate of lack of access or difficulty in handling technology in the present study.

Nearly 70% of the otorhinolaryngologists who participated already used telemedicine in their daily lives, highlighting the relevance that such a tool has in the field. However, the use of such instruments is much lower when we compare the private and public sectors. A total of 84% of the respondents had never conducted a consultation through the Brazilian Unified Health System (Sistema Único de Saúde, SUS, in Portuguese) using this technology. The cause of this discrepancy should be investigated to develop public policies that can be implemented to improve this scenario.

According to the present study, the digital tools most used for consultations are not specific telemedicine platforms, but communication applications such as Whatsapp and Zoom. Despite the encrypted security of these platforms, they were not created for medical use; therefore, they do not provide a secure location to write electronic medical records. 10 The present study did not evaluate whether doctors record their teleconsultations on other electronic medical platforms. Therefore, this evaluation should be carried out in subsequent studies, since every medical consultation must be duly registered.

Telemedicine seems to be widely used in otorhinolaryngology for both initial consultations and follow-ups. A total of 90% of physicians in the present study agree that the lack of otorhinolaryngological physical examination is a problem. It is evident that most otorhinolaryngological and cervicofacial diseases involve the need for physical examination, which is undoubtedly one of the main flaws of telemedicine. 8 9 This assertion is corroborated when we note that 25% of physicians who do not use the tool blame the absence of physical examination for their decision. The authors wonder if this barrier can be overcome by new technologies or if this difficulty will always be associated with virtual consultations.

A total of 64% of otorhinolaryngologists reported that they solve their patients' problems frequently or very frequently through telemedicine, which draws attention to the high rate of resolution of this method even in early stages. This indicates that the regulation of this tool during the pandemic in Brazil was correct, as it may have contributed to reducing viral transmission. Most physicians in the present study (75%) intend to maintain tele-appointments even after the end of the pandemic. Considering the aforementioned information, we emphasize the importance of new studies in the field with the objective of increasing the quality of mapping the use of these new technologies. It is evident that the use of these tools should be maintained and possibly increase. For this phenomenon to occur in an orderly manner, the otorhinolaryngological community must remain vigilant so that this technology is properly used, protecting patients and physicians.

## Conclusion

The use of telemedicine is witnessing a rapid expansion, and the present study underscores the involvement of Brazilian otolaryngologists in this trend. Nevertheless, certain barriers may hinder the use of this tool. We emphasize that additional studies are required to comprehensively apprehend the role that telemedicine will play in the field of otolaryngology in the coming years.
